# Network Pharmacology-Based Approach Combined with Bioinformatic Analytics to Elucidate the Potential of Curcumol against Hepatocellular Carcinoma

**DOI:** 10.3390/genes13040653

**Published:** 2022-04-07

**Authors:** Xufeng Huang, Hafiz Muzzammel Rehman, Attila Gábor Szöllősi, Shujing Zhou

**Affiliations:** 1 Faculty of Dentistry, University of Debrecen, 4032 Debrecen, Hungary; huangxufeng@mailbox.unideb.hu; 2 Alnoorians Group of Institutes 55-Elahi Bukhsh Park, Amir Road, Shad Bagh, Lahore 54000, Pakistan; muzzammel.phd.ibb@pu.edu.pk; 3 School of Biochemistry and Biotechnology, University of the Punjab, Lahore 54590, Pakistan; 4 Department of Immunology, University of Debrecen, 4032 Debrecen, Hungary; 5 Faculty of Medicine, University of Debrecen, 4032 Debrecen, Hungary; shujing.zhou@mailbox.unideb.hu

**Keywords:** network modeling, drug discovery, data integration

## Abstract

Purpose: Modern, open-source databases provide an unprecedented wealth of information to help drug development. By combining data available in these databases with the proper bioinformatical tools, we can elucidate the molecular targets of natural compounds. One such molecule is curcumol, a guaiane-type sesquiterpenoid hemiketal isolated from Rhizoma Curcumae, which is used for a broad range of diseases in traditional Chinese and Indian medicine. It has been reported to exert anti-tumor activity, but the intrinsic molecular mechanism in hepatocellular carcinoma (HCC) is unclear. Therefore, the present study was designed to reveal the predictive targets and biological mechanisms of curcumol against HCC via a network pharmacology-based approach combined with bioinformatic analytics and to provide proof of concept for further similar investigations. Methods: Data available from open-source databases (Traditional Chinese Medicine Systems Pharmacology, Comparative Toxicogenomic Database, The Cancer Genome Atlas, the Human Protein Atlas project) was processed with the help of a variety of open-source tools (SwissADME, SwissTargetPrediction, JVenn, Gene Ontology, Kyoto Encyclopedia of Genes and Genomes, GeneMANIA, Cytoscape). Results: In the present study, the potential of curcumol against HCC was unraveled by network pharmacology-based elucidation. It suggests that curcumol shows exciting druggability with 44 potent homo sapiens biotargets against HCC. The GO terms and KEGG pathways enrichment analyses, curcumol-targets-pathways-HCC network, PPI network, and corresponding in-depth topological analyses, as well as survival analysis, molecular docking simulation indicate that the potential mechanism of curcumol against HCC is complicated, as it may act in various ways, mainly by inducing apoptosis and modulating the inflammatory response, increasing presentation of HCC-specific protein. Conclusion: The present study highlights the potential of curcumol against HCC, giving reference to further experimental study. It also presents a roadmap that can be followed to conduct in silico prescreening of other compounds of interest.

## 1. Introduction

Hepatocellular carcinoma (HCC) is the most common type of primary liver cancer that occurs most often in people with long-term liver diseases, especially cirrhosis caused by hepatitis viral infection [1].

Natural products from traditional herbal medicines are precious sources for new drug discovery. Curcumol, as one of the typical examples, is a bioactive extract of essential volatile oils from Curcumae Rhizoma [2], which has been used for centuries as an essential ingredient in traditional herbal formulas in the treatments of various diseases (e.g., blood stasis, alleviating pain, and more) [3]. Current studies suggest that curcumol exerts tremendous therapeutic effects against tumors, reducing inflammation, and protecting the liver [4,5,6,7,8]. These findings hint that curcumol may serve as a potential new drug against HCC. However, this possibility is inadequately studied; therefore, the present study attempts to make a critical first step in this field via a network pharmacology-based approach.

We first conducted an “absorption, distribution, metabolism, and excretion” (ADME) evaluation by using the Traditional Chinese Medicine Systems Pharmacology (TCMSP) database and SwissADME analysis platform to investigate the druggability of curcumol at the molecular level.

Then, putative targets of curcumol were mined from Comparative Toxicogenomic Database (CTD) and SwissTargetPrediction server. Meanwhile, we obtained the therapeutic targets of HCC from The Cancer Genome Atlas (TCGA) cohort and Genotype-Tissue Expression (GTEx) portal, GeneCards database, and Online Mendelian Inheritance in Man (OMIM) database. An intersection containing all the common targets of the putative targets of curcumol and the therapeutic targets of HCC was calculated by the JVenn program.

Gene Ontology (GO) terms and Kyoto Encyclopedia of Genes and Genomes (KEGG) pathways analyses were carried out by WEB-based GEne SeT AnaLysis Toolkit (WebGestalt) for these common targets to gain a macroscopic understanding of their functions.

Moreover, a preliminary protein-protein interaction (PPI) network was constructed by the GeneMANIA plug-in of Cytoscape software. To have a deeper insight into the network, a cluster classification of the preliminary PPI network was performed by the MCODE plug-in of Cytoscape software. Each cluster was followed by GO terms and KEGG pathways analyses. In addition, by using the CytoNCA plug-in of Cytoscape software, a core PPI subnetwork with 9 key nodes and 22 edges was extracted.

Moreover, a survival analysis was also performed for the common targets, where we found 17 significantly prognosis-related genes from various types of survival.

By using the JVenn program again, we sorted out two key targets (i.e., RELA and SQSTM1) from the core PPI subnetwork that are significantly prognosis related. We then, from the aspects of cancer grading and Spearman correlation analysis, provided a brief summary of RELA and SQSTM1 to show their importance in HCC. Furthermore, with the help of the Human Protein Atlas project, we clinically verified their importance in HCC patients by comparing the expression in normal liver tissue and pathological liver tissue. Consequently, it was found that they themselves are positively correlated, and their overexpression is associated with HCC carcinogenesis.

Finally, molecular docking and molecular dynamic simulation were performed to investigate their binding to the curcumol molecule.

**Figure 1:**
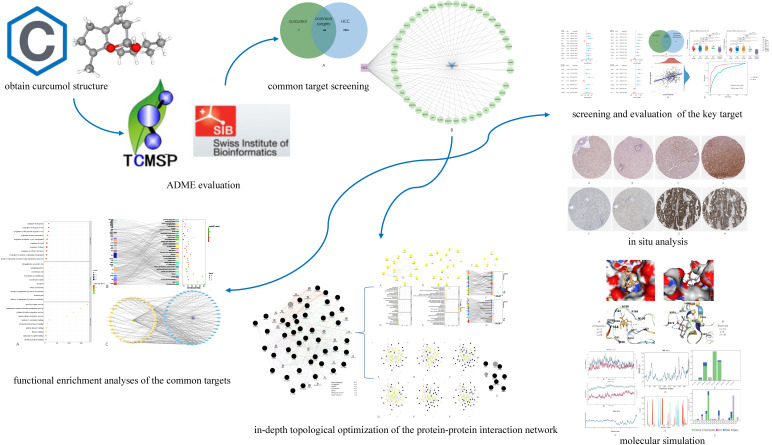
Workflow of the present study in a graphical manner.

Figure 1 below demonstrates the workflow of the present study in a graphical manner.

## 2. Materials and Methods

### 2.1. ADME Evaluation

SwissADME server (http://www.swissadme.ch/, accessed on 22 November 2021) is an online tool that offers structure based ADME parameter prediction. In the present study, given the three-dimensional structure of the curcumol molecule, it was first used to roughly assess its druggability. Then, we searched with the keyword “Curcumol” on the TCMSP database (https://tcmsp-e.com, accessed on 22 November 2021) to further confirm the pharmacological properties in exact values in a literature-based manner [9,10,11,12].

### 2.2. Common Targets Mining

#### 2.2.1. Identification of the Putative Targets of Curcumol

The Comparative Toxicogenomic Database (CTD; http://ctdbase.org/, accessed on 22 November 2021) is a publicly available digital ecosystem that relates toxicological information on chemicals, genes, phenotypes, and diseases, to literature data [13]. In the present study, the putative targets of curcumol were first sorted out of the literature pool and then manually filtered to ensure they were homo sapiens biotargets.

SwissTargetPrediction (http://www.swisstargetprediction.ch/, accessed on 22 November 2021) is a structure-based target predictor [10,11,12]. The three-dimensional structure of curcumol was downloaded from the PubChem database (https://pubchem.ncbi.nlm.nih.gov/, accessed on 22 November 2021) [14,15] and input into the searching box.

#### 2.2.2. Identification of the Therapeutic Targets of HCC

Raw counts of RNA-sequencing data (level 3) and corresponding clinical information were obtained from the TCGA cohort (https://portal.gdc.cancer.gov/, accessed on 22 November 2021) [16]. Normal healthy tissue data were obtained from the GTEx portal (https://gtexportal.org/home/, accessed on 22 November 2021) [17] as part of the control group. All the mentioned data were analyzed by R packages “limma” in R foundation (v.4.0.3) on Scihub (https://www.aclbi.com, accessed on 22 November 2021) for statistical computing [18]. In addition, the GeneCards database (https://www.genecards.org/, accessed on 22 November 2021) [19] and OMIM database (https://www.omim.org/, accessed on 22 November 2021) [20] were also searched.

#### 2.2.3. Common Target Screening

The JVenn program (http://jvenn.toulouse.inra.fr/app/index.html, accessed on 22 November 2021) is a JavaScript-based program that can exert a Venn diagram of up to 6 data sets [21]. In the present study, it was run to calculate the intersection of the data sets obtained from steps 2.1 and 2.2. Then, we visualized the expression of the distribution of each target in HCC by integrating the data acquired from the TCGA cohort and elucidated the regulation of the expression of common targets in HCC at the gene set level by conducting gene set enrichment analysis (GSEA) in Gene Set Cancer Analysis (GSCA) online platform (http://bioinfo.life.hust.edu.cn/GSCA/, accessed on 22 November 2021) [22].

### 2.3. Functional Enrichment Analyses and Curcumol-Targets-Hcc-Pathways Network Construction

To gain a macroscopic understanding of the functions of common targets, we carried out GO terms and KEGG pathway enrichment analyses by WebGestalt (http://www.webgestalt.org/, accessed on 22 November 2021) [23]. The results were then further visualized by the SRPlot online toolkit (http://www.bioinformatics.com.cn/en, accessed on 22 November 2021). Consequently, by using Cytoscape software, we constructed a curcumol-targets-HCC-pathways network [24].

### 2.4. Ppi Network Construction and In-Depth Structural Optimization

#### 2.4.1. Preliminary PPI Network Construction

The GeneMANIA plug-in of Cytoscape software is a robust application that can analyze and prioritize functional assays [25]. Given query genes, GeneMANIA will list proteins with shared properties and similar functions as a PPI network. In the present study, the common targets were typed into the search bar, and the homo sapiens option was selected in the filter button.

#### 2.4.2. Functional Clustering

After obtaining the preliminary PPI network, MCODE, a Cytoscape plug-in, was used to cluster the protein network [26]. The parameter “K-Core” value was set to be 3, and the other parameters were the same as default (i.e., degree cutoff: 2, cluster finding: haircut, node score cutoff: 0.2, max. depth: 100). Each cluster creates a subnetwork to display separately, followed by GO terms and KEGG pathways enrichment analyses.

#### 2.4.3. Core PPI Subnetwork Extraction

CytoNCA, another Cytoscape plug-in that is capable of network centrality analysis, was used to extract the core PPI subnetwork via in-depth topological analyses [27]. We first set the filtering criteria to be above the average of closeness, betweenness, network, eigenvector, LAC, and degree centralities of the preliminary network to extract the corresponding subnetworks, respectively. Then, we used the intersectional merge function of the Cytoscape software to isolate the core PPI subnetworks from them. Thereafter, we sorted out the key nodes in the preliminary PPI network (i.e., the nodes in the core PPI subnetwork).

### 2.5. Survival Analyses

To identify the targets that were prognosis related in HCC, a total of 44 common targets underwent survival analysis, including overall survival (OS), progression-free survival (PFS), disease-free survival (DFS), and disease-specific survival (DSS) through R packages “ggplot2” and “survival” on Scihub [28,29]. The *p* values and hazard ratio (HR) with a 95% confidence interval (CI) were generated by log-rank tests and univariate COX proportional hazards regression. The results were plotted as forest plots and Kaplan–Meier (KM) curves.

### 2.6. Screening of the Key Targets and Exploration of Their Expression and Correlation in Hcc

By using JVenn program to calculate the intersection of the 9 key nodes from step 4.3 and the 17 significantly prognosis-related genes from step 5, 2 key targets (i.e., RELA and SQSTM1) were found.

In order to unravel the importance of the key targets, we conducted Kruskal–Wallis test to compare their expression levels in various cancer grades (i.e., G1, G2, G3, G4) and normal tissues. Afterward, we performed a receiver operative characteristic (ROC) analysis to see if RELA and SQSTM1 are eligible to be the biomarker in HCC. Tumoral RNA-seq data were downloaded from the TCGA cohort, while data of normal tissue samples were from the GTEx portal. We also used the Spearman method to describe the correlation between RELA and SQSTM1. All the statistical analyses were performed by R packages “ggstatsplot” and “pROC” on SciHub, where *p* < 0.05 was considered statistically significant [30,31].

### 2.7. In Situ Analysis

At this step, by using the Human Protein Atlas project (https://www.proteinatlas.org/, 26 November 2021), we inspected the histological slides of HCC patients and normal individuals [32]. Selected slides that are demonstrated in the present study include 2 normal liver tissue samples and 2 pathological liver tissue samples for both identified markers (HPA068843 and CAB004587 were used to stain SQSTM1, while CAB004264 and CAB005030 were used to stain RELA). Images are available at the following URL: https://www.proteinatlas.org/ENSG00000173039-RELA 26 November 2021 and https://www.proteinatlas.org/ENSG00000161011-SQSTM1 26 November 2021.

### 2.8. Molecular Docking of Key Targets

To simulate the interaction of the key targets with the curcumol molecule, structures of the key targets from PDB (RELA ID: 1nfi, https://www.rcsb.org/structure/1NFI, 28 November 2021) were acquired and prepared in PyMOL (v1.2r3pre) by removing water molecules, adding charge, and parameterizing for molecular docking on AutoDock Vina-based platform, CBdock (http://clab.labshare.cn/cb-dock/php/, 28 November 2021) [33,34,35,36]. In this way, we obtained their corresponding affinity energy values and specific three-dimensional coordinates. Notably, as there was no entire STSQM1 structural file available, we used an Alphafold document (SQSTM ID: AF-Q13501-F1-model, https://alphafold.ebi.ac.uk/entry/Q13501, 28 November 2021) for docking [37,38].

### 2.9. Molecular Dynamic Simulation

Molecular dynamics simulations were performed to investigate the binding conformational stability of the protein-ligand complex. The stability of the protein-ligand complex was maintained during 100 ns simulations using Desmond software for compounds based on root mean square deviation (RMSD), root mean square fluctuation (RMSF), and hydrogen bond interactions [39,40].

## 3. Results

### 3.1. ADME Evaluation

During ADME evaluation, multiple pharmacological parameters are discussed (e.g., MW, Hdon, Hacc, RBN, OB, etc.). MW (molecular weight) refers to the mass of a molecule, Hdon and Hacc refer to hydrogen bond donor and receptor, AlogP value represents the partition coefficient between octanol and water [41]. They are the parameters of Lipinski’s “rule of five”, which is the most common empirical rule to assess if a chemical is suitable to be developed into orally delivered drugs. RBN is the number of free self-rotationable bonds (i.e., any single bond, not in a ring, bound to a nonterminal heavy atom, excluding the C-N bond) [42]. It reflects the flexibility of the molecule. A molecule with a 10 or even smaller RBN value is predicted to have suitable oral bioavailability. OB (oral bioavailability) is the percentage of the dose of a drug that is orally administrated and reaches the systemic circulation system without changing any of it [43]. A high OB value is often the key to determining whether a bioactive molecule is suitable to serve as an orally administrated therapeutic agent. DL (drug-likeness) is a qualitative concept to estimate how “drug-like” a prospective compound is [44]. Caco2 permeability (Caco2) is the passive diffusion of drugs across intestinal epithelium (i.e., to be more exactly speaking, in the case of human intestinal cell line “Caco2”), which represents the efficiency of drug intake in the gut [45]. BBB is an abbreviation of “blood-brain barrier”. Here, its value is a quantified tool for researchers to measure how well a molecule of interest can penetrate the blood-brain barrier. Usually, BBB < −0.3 is non-penetrating (BBB-), −0.3 < BBB < +0.3 is moderate penetrating (BBB±), and BBB > 0.3 is strong penetrating (BBB+) [46]. TPSA is the abbreviation of “the polar surface area” of a molecule. It is defined as the surface sum over all polar atoms, including their attached hydrogen atoms [47]. TPSA > 140Å^2^ usually means the molecule is poor at penetrating the cell membrane, while if TPSA < 60 Å^2^, usually it is suitable at penetrating the cell membrane.

As an official recommendation by the TCMSP database, suggested drug screening criteria usually should be RBN < 10, OB ≥ 20%, DL ≥ 0.1, and TPSA < 60 Å^2^. From this aspect, as the curcumol molecule possesses the following parameters: OB = 103.55%, DL = 0.13, TPSA = 0.25 Å^2^, RBN = 1, it’s deemed to possess exciting druggability (detailed ADME values are available at Table 1).

As a supplementary assessment (Files S1–S7), the “Lipinski’s rule of five” (i.e., suitable absorption is more likely when MW < 500 Da, AlogP < 5, Hdon < 5, Hacc < 10), was applied [48]. According to this rule, as the curcumol molecule possesses the following parameters: MW = 236.39, AlogP = 2.79, Hdon = 1, Hacc = 2, it is considered as a promising candidate for future drug optimization.

### 3.2. Common Targets Mining

According to the search results, in total, 51 putative targets of curcumol molecules were found. Their gene IDs and official gene names are given in Supplementary Material Table S1. For HCC, 7958 targets were identified from multiple open-accessible human genomic platforms (TCGA-GTEx, GeneCards, OMIM, etc.). After deleting the repeated subjects, we created a list containing 7958 potential therapeutic targets of HCC.

**Figure 2:**
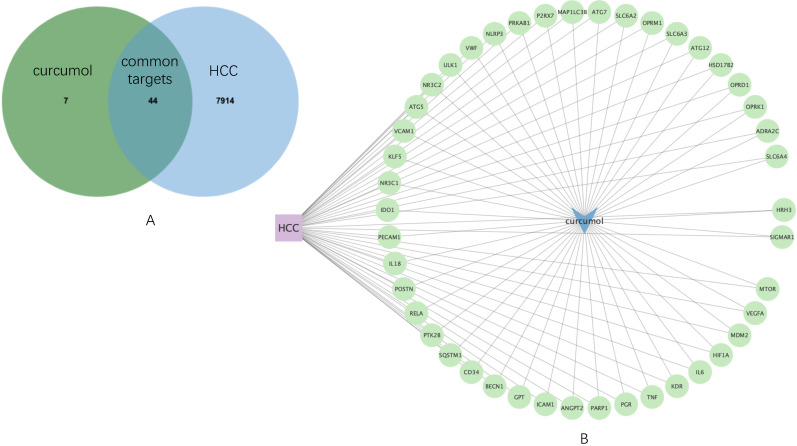
Common targets mining and their expression distribution. (**A**) Venn diagram shows 44 common targets of curcumol and HCC. (**B**) HCC-curcumol-common targets network.

Then, by using JVenn, 44 common targets were sorted (Figure 2A), which was used to construct an HCC-curcumol-common targets network by Cytoscape software (Figure 2B). To gain a more comprehensive understanding of the expression of the common targets in HCC, their expression distribution of each gene in HCC patients and healthy individuals as a comparison were screened in Supplementary Material File S1. In addition, we conducted gene set enrichment analysis (GSEA) for the common targets, in which we found the enrichment score is 0.44 and the normalized enrichment score (NES) is 1.05, indicating that the common targets as a gene set, the expression is upregulated in HCC (details can be found in Supplementary Material File S2).

This section may be divided into subheadings. It should provide a concise and precise description of the experimental results, their interpretation, as well as the experimental conclusions that can be drawn.

### 3.3. Functional Enrichment Analyses and Curcumol-Targets-Hcc-Pathways Network Construction

**Figure 3:**
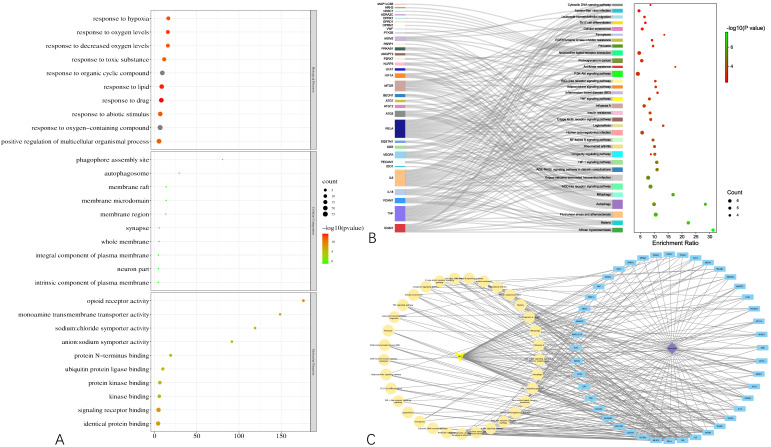
Results of functional enrichment analyses of the common targets and curcumol-targets-pathways-HCC network. (**A**) Bubble plot demonstrating top 10 most enriched GO terms in biological process, cellular component, and molecular function. (**B**) Bubble plot combined with Sankey diagram demonstrating statistically significant KEGG pathways and the genes within each pathway. (**C**) Curcumol-targets-pathways-HCC network.

To gain a macroscopic understanding of the functions of the common targets, GO terms and KEGG pathways enrichment analyses were carried out. Consequently, the top 10 most enriched GO terms in biological processes, cellular components, and molecular functions were screened in the form of a bubble plot. As shown in Figure 3A, the most enriched GO term in a biological process is “response to oxygen-containing compound”, followed by “response to organic cyclic compound”, and then “response to drug”, while the most enriched GO term in cellular component and molecular function is “whole membrane” and “opioid receptor activity”, respectively. For KEGG pathways analysis, 36 pathways that are statistically significant (FDR < 0.05) were screened in the form of a bubble plot as well but combined with a Sankey diagram, which demonstrates the genes within each pathway. As shown in Figure 3B, these pathways are mainly related to apoptosis (e.g., hsa04136 and hsa04140 autophagy, hsa04137 mitophagy), inflammation (e.g., hsa04621 Nod-like receptor signaling pathway) and responses to viral infection (e.g., hsa05167 Kaposi sarcoma-associated herpesvirus infection). Notably, through the Sankey diagram, it was found that RELA participates in the largest number of pathways, followed by IL6 and TNF.

Based on the results of the functional enrichment analyses, a curcumol-targets-pathways-HCC network was constructed, as shown in Figure 3C, which contains 79 nodes and 265 edges. Among these nodes, RELA, the proto-oncogene that transcripts p65, has the highest degree in the network, followed by TNF, IL6, MTOR, which are responsible for cell growth and inflammation.

### 3.4. PPI Network Construction and In-Depth Structural Optimizationtion

**Figure 4:**
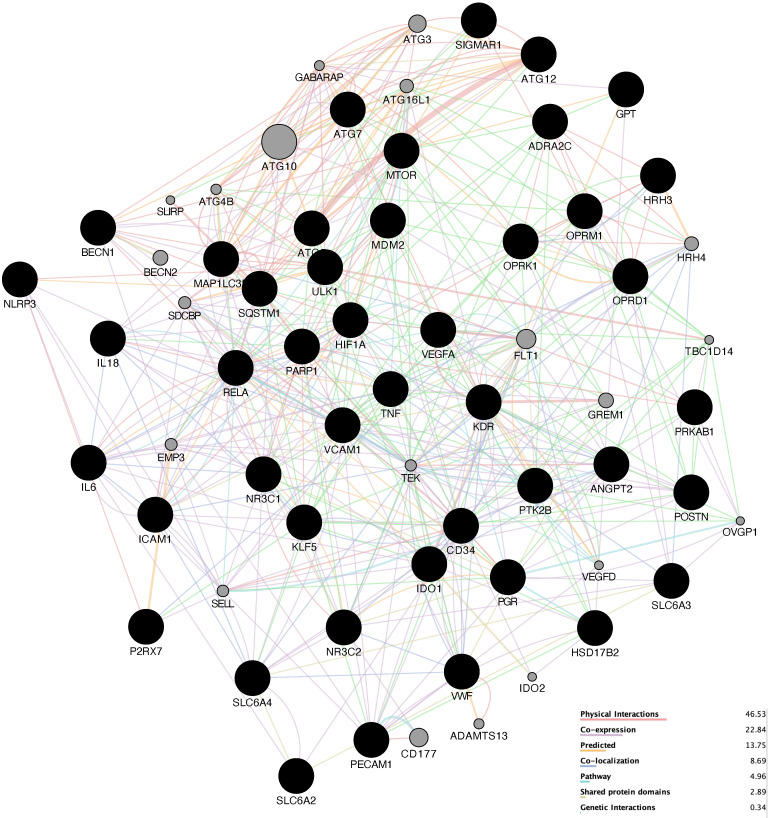
Preliminary PPI network. The higher the degree of the node, the larger the node and the darker the color of the node. The stronger interaction between the nodes, the thicker and deeper color of the edge.

Attempting to reveal the underlying potential therapeutic mechanism against HCC, we used GeneMANIA to build a preliminary PPI network with 64 nodes and 512 edges, as shown in Figure 4. The network density is 0.191, the heterogenicity is 0.443, and the centrality is 0.212.

**Figure 5:**
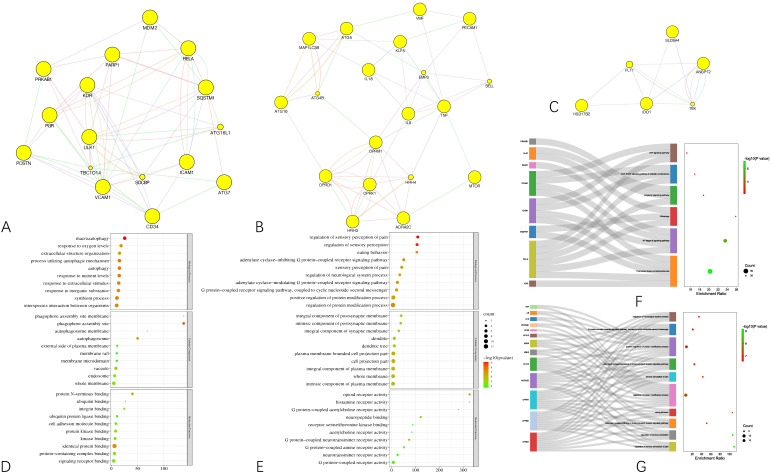
Cluster classification of the preliminary PPI network. (**A**−**C**) are clusters 1 to 3 divided through the MCODE plug-in, respectively. (**D**,**E**) Bubble plots demonstrating the top 10 most enriched GO terms in biological process, cellular component, and molecular function of cluster 1 and cluster 2, respectively. (**F**,**G**) Bubble plots combined with Sankey diagrams demonstrating the KEGG pathways with statical significance (FDR < 0.05) and the proteins within each pathway of cluster 1 and cluster 2, respectively.

Proteins tend to perform their corresponding functions under in vivo conditions by forming different functional clusters. Therefore, we used the MCODE plug-in to analyze the preliminary PPI network to identify important functional clusters to better understand the underlying mechanisms. In total, there were 3 clusters classified as shown in Figure 5A−C, in which cluster 1 subnetwork processes 16 nodes and 58 edges with a score of 6.133, cluster 2 subnetwork possesses 19 nodes and 58 edges with a score of 5.444, and cluster 3 subnetwork possesses 6 nodes and 14 edges with a score of 4.000.

Each cluster was followed by GO terms and KEGG pathways enrichment analyses. The results were integrated into Figure 5D,E. Notably, due to the lack of statistical significance (FDR < 0.05), we could not conduct such analyses for cluster 3. Results show that proteins in cluster 1 are mainly associated with apoptosis, while proteins in cluster 2 are mainly associated with neurological signaling.

Besides the previous analyses, a topological analysis of the preliminary PPI network is also necessary. Therefore, the CytoNCA plug-in was used to find the core proteins that frame the preliminary network.

First, above all, we calculated the average value of degree, betweenness, closeness, eigenvector, LAC, network centralities of the preliminary network, which is 12, 64.3125, 0.543188007, 0.11156333, 4.789801879, and 6.193579598, respectively.

**Figure 6:**
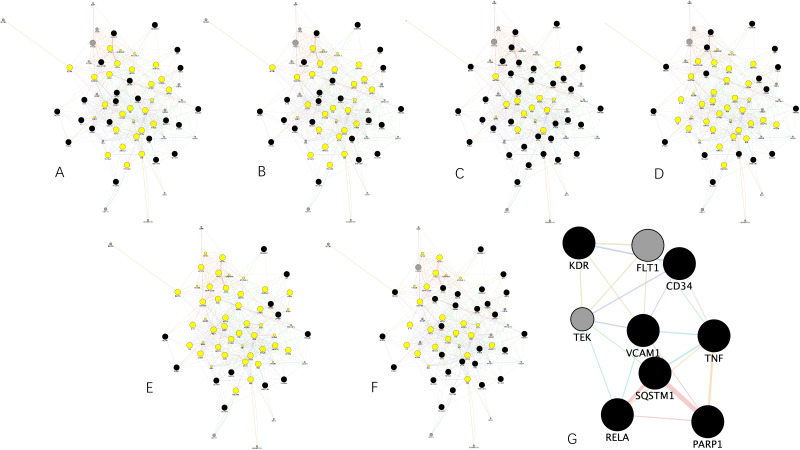
The process of core PPI subnetwork generation. (**A**–**F**) PPI subnetworks demonstrating the subnetworks extracted from the preliminary PPI network according to the closeness, betweenness, network, eigenvector, LAC, and degree centrality method, respectively. (**G**) Core PPI subnetwork generated by intersectional merge of (**A**–**F**) PPI subnetworks.

Then, we extracted the corresponding networks by setting the threshold as these averages. That is to say, in other words, according to the above-mentioned averages, the nodes in the preliminary PPI network greater than these averages were sorted out for corresponding subnetwork construction. Figure 6A−F demonstrates the subnetwork extracted through degree, betweenness, closeness, eigenvector, LAC, and network centrality method, respectively.

Furthermore, we extracted a core PPI subnetwork by using the intersectional merge function of the Cytoscape software. In this way, common nodes showing up within the above-mentioned subnetworks were sorted out for core PPI subnetwork construction. As a result, a core PPI subnetwork in Figure 6G containing 9 key nodes (i.e., KDR, TEK, RELA, SQSTM1, VCAM1, FLT1, CD34, TNF, and PNRP1) and 22 edges was extracted. Through the figure, it can be clearly seen that SQSTM1 shares the strongest and the greatest number of connections with the others, followed by RELA.

### 3.5. Survival Analysis

By considering *p* < 0.05 as statistically significant, we conducted log-rank tests and univariate Cox proportional hazards regression for all the 44 common targets to carry out survival analysis, including overall survival (OS), progression-free survival (PFS), disease-free survival (DFS), and disease-specific survival (DSS). In total, 17 genes were identified as prognosis related in HCC in case of different types of survival.

**Figure 7:**
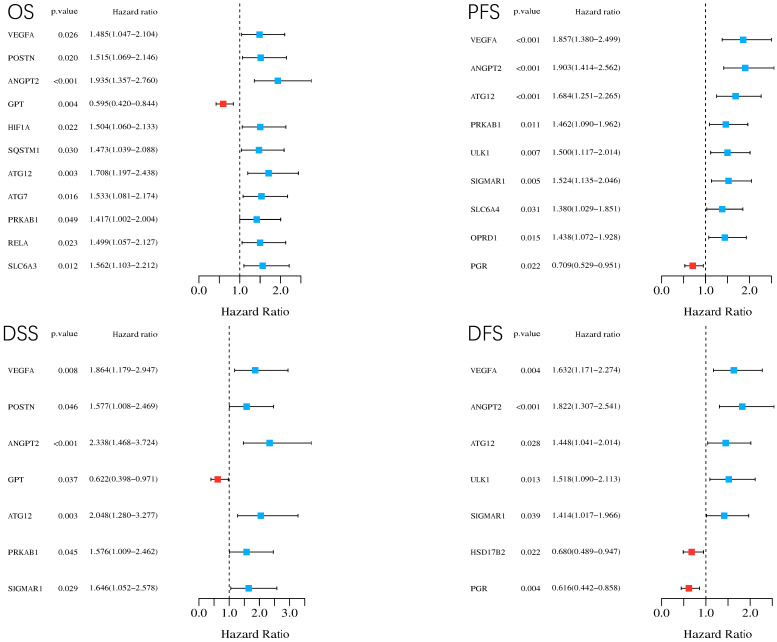
Forest plots demonstrating statistically significant genes and their *p* values, hazard ratios (HR) with 95% confidence interval (IC) in different types of survival (i.e., OS, PFS, DFS, and DSS). OS: overall survival; PFS: progression-free survival; DFS: disease-free survival; DSS: disease-specific survival. HR > 1 indicates that the corresponding gene is a protective factor, while HR < 1 indicates the corresponding gene is positively associated with the deterioration of cancer. When *p* < 0.05, the line of the gene will not touch the reference vertical line in the middle of the forest plot, meaning that this gene is statistically significant.

Their corresponding KM curves are available in Supplementary Material File S7. The forest plots in Figure 7 demonstrate statistically significant genes and their *p* values, hazard ratios (HR) with 95% confidence interval (IC) in different types of survival (i.e., OS, PFS, DFS, DSS). In summary, through the survival analysis, except GPT, PGR, and HSD17B2, the rest (i.e., VEGFA, ANGPT2, ATG12, PRKAB1, ULK1, SIGMAR1, SLC6A4, OPRD1, AGT7, HIF1A, POSTN, SLC6A3, GPT, RELA, and SQSTM1) are related to poor prognosis.

### 3.6. Screening for the Key Targets and Exploration of Their Expression and Correlation in HCC

**Figure 8:**
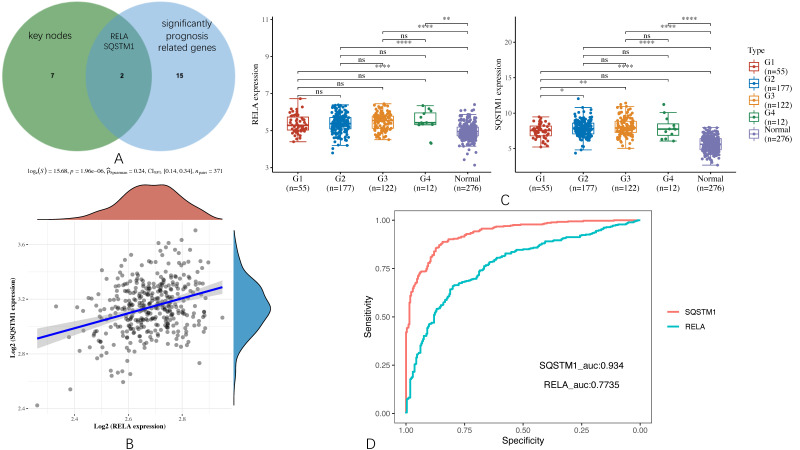
Screening for the key targets and their brief summary. (**A**) Venn diagram that screens the key targets (i.e., RELA, SQSTM1). (**B**) The correlations between RELA and SQSTM1 analyzed with the Spearman method. The density curve on the right represents the trend in the distribution of SQSTM1 expression in 371 HCC samples from the TCGA cohort. The upper density curve represents the trend in the distribution of RELA expression in 371 HCC samples from the TCGA cohort. (**C**) The expression levels of RELA and SQSTM1 in various cancer grades (i.e., G1–G4) and normal tissue as comparison. The number of the stars indicates the statistical significance (*: *p* < 0.05, **: *p* < 0.01, ****: *p* < 0.0001). ns means non-significant. (**D**) Diagnostic ROC curve for RELA and SQSTM1 in HCC.

By using the JVenn program, two key targets, RELA and SQSTM1, were sorted out from the intersection of the nine key nodes and the significantly prognosis-related genes, as shown in Figure 8A. Then, we explored the correlation between the expression of RELA and SQSTM1 through the 371 samples collected from the TCGA cohort. The result is shown in Figure 8B indicates that RELA and SQSTM1 are positively associated with the Spearman coefficient > 0 and the corresponding *p*-value < 0.001. This hints that they have potential synergistic effects.

Moreover, we also explored their expression levels at different HCC grades and normal tissues by Kruskal–Wallis’s test. The result is shown in Figure 8C suggests that their expression levels are not significantly related to different cancer grades, but there is a huge difference between cancer and normal tissues.

In order to verify whether RELA and SQSTM1 may serve as reliable biomarkers in HCC, we further performed a diagnostic receiver operative characteristic (ROC) analysis, as shown in Figure 8D, in which we followed the philosophy of the gold-standard test (i.e., dichotomization of the mentioned samples in TCGA cohort and GTEx portal into disease present, which includes all tumor grades from G1 to G4, or disease absent meaning that the sample is normal) [49,50]. It was found that the area under curve (AUC) of RELA is 0.7735, and that of SQSTM1 is 0.934. As generally speaking, the AUC value was considered excellent if it is from 0.9 to 1, good if it is from 0.8 to 0.9, fair if it is from 0.7 to 0.8; herein, the reliability of RELA should be thought of as fair, and that of SQSTM1 should be thought of as excellent [51].

### 3.7. InSitu Analysis

**Figure 9:**
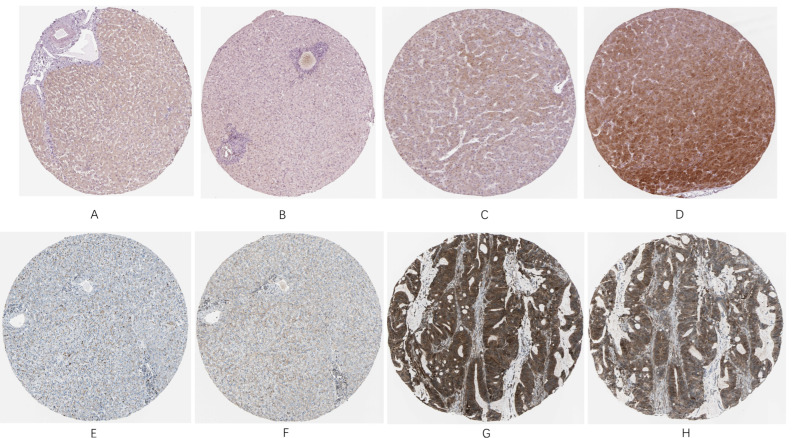
(**A**,**B**) are normal histological slides from liver tissue sample of a female patient at age 50, (**C**,**D**) are pathological histological slides from liver tissue sample of a male patient at age 67, (**E**,**F**) are normal histological slides from liver tissue sample of a female patient at age 32, (**G**,**H**) are pathological histological slides from liver tissue sample of a female patient at age 73.

In order to verify the importance of RELA and SQSTM1 proteins in HCC patients, we compared their expression with that of normal individuals. Both proteins showed relatively low expression in normal tissues, while in the histological slides from HCC patients, they exhibited high expression levels, as shown in Figure 9A–H. Therefore, it is verified clinically that they are important poor prognosis-related biomarkers.

### 3.8. Molecular Docking of Key Targets

We downloaded the corresponding three-dimensional structures from RCSB PDB to conduct blind docking. The docking site was set in a cubic box in the center of the initial ligand. The results of RELA docking indicates that its binding with curcumol possesses two hydrogen bonds with ARG 187 and ALA 188 and two hydrophobic interactions with ASP 277 and ASN 190, while the results of SQSTM1 docking indicates that its binding with curcumol possesses two hydrogen bonds with ARG 187 and ALA 188 and two hydrophobic interactions with ASP 277 and ASN 190.

**Figure 10:**
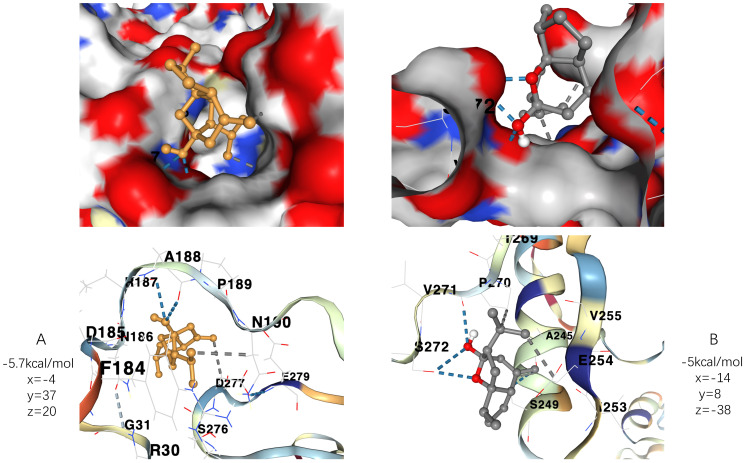
Molecular docking results with affinity energy and specific three-dimensional coordinates. (**A**) demonstrates the three-dimensional conformation of the docking of RELA protein with curcumol molecule and the corresponding binding affinity. (**B**) Demonstrates the three-dimensional conformation of the docking of SQSTM1 protein with curcumol molecule and the corresponding binding affinity.

Moreover, as the more negative the docking score is, the higher the binding force is between the compound and the protein, we, therefore, found the best run provided the binding affinity of −5.7 kcal/mol and −5 kcal/mol to RELA and SQSTM1, respectively, taking together with their specific interactions into account. As generally speaking, affinity energy ≤ −5 kcal/mol is deemed as high affinity. RELA and SQSTM1 thus can be considered as key players that participate in the potential mechanism against HCC of curcumol. The docking results can be viewed in a three-dimensional manner in Figure 10A,B.

### 3.9. Molecular Dynamic Simulation

**Figure 11:**
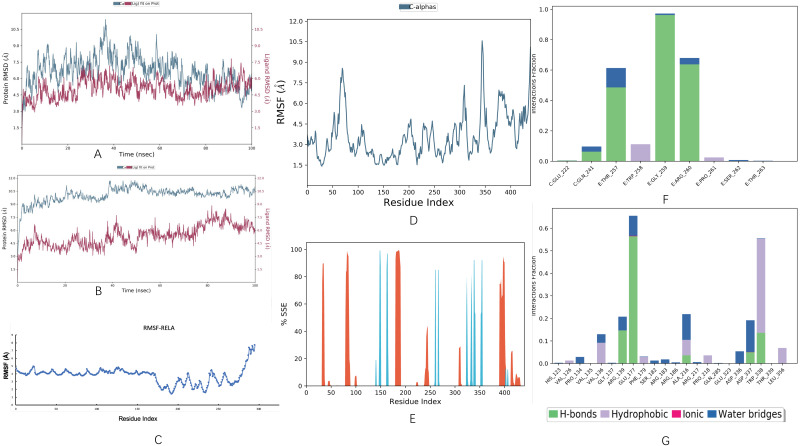
Molecular dynamic simulation performed on the top hits containing high binding energies. (**A**,**B**) Root mean square deviation (RMSD) plot of curcumol-RELA complex and curcumol-SQSTM1 complex, respectively. (**C**,**D**) Root mean square fluctuation (RMSF) plot of RELA and SQSTM1, respectively. (**E**) Protein secondary structure elements (SSE) of SQSTM1. (**F**,**G**) protein-ligand contacts of curcumol-RELA complex and curcumol-SQSTM1 complex, respectively.

Molecular dynamics simulations were performed on the top hits containing high binding energies. Over the simulation period, the projected conformational changes from the initial structure were presented in RMSD. Moreover, structural stability, atomic mobility, and residue flexibility at times of interaction of protein-hit were expressed with RMSF values. The peaks of the RMSF graph represent the fluctuation portion of the protein through the simulation. The N- and C-terminals show more changes than any other portion of the protein. α helices and β strands show less fluctuation, as they are stiffer than the unstructured part of the protein than the loop portion. The RMSD of the curcumol-RELA complex showed deviation at almost 30 ns for 2 Å, and then the system was equilibrated throughout the simulation, as shown in Figure 11. The RMSD of the curcumol-SQSTM1 complex showed little deviation, but it was in the range of 3 Å. In general, the complex was stable throughout the simulation, the ligand remained inside the binding pocket and made important interactions, and the backbone was consistent. The deviation might be due to conformational changes, as shown in Figure 11B. The RMSF for RELA showed little fluctuation throughout the simulation, as shown in Figure 11C. Similarly, in RMSF of SQSTM1, there was fluctuation near ASP 69 to ALA 76, GLU 309 to LYS 313, and LYS 344 to ASP 347 residues, and the remaining structure was comparatively stabilized, and there was not much fluctuation where ligand made contacts with protein, as shown in Figure 11D. The fluctuation in the protein might be due to 83% of the unstructured part of the protein. Furthermore, secondary structure elements (SSE) such as α helices and β strands were monitored throughout the simulation. It turned out to be 8.38% of helix, 4.60% of strands, and 12.98% of total SSE in the SSE analysis, as shown in Figure 11E.

The curcumol-RELA complex possessed several different types of interactions, including hydrogen bonds, water bridges, and hydrophobic, as shown in Figure 11F, while in the case of the curcumol-SQSTM1 complex, the types of intermolecular interactions during the entire simulation were found to be more diverse, including hydrogen bonds, ionic, water bridges, and hydrophobic. The residues participating in these interactions include VAL 126, PRO 134, VAL 136, ARG 139, GLU 177, PHE 179, SER 182, SER 183, ALA 216, PRO 218, GLU 323, ASP 336, ASP 337, TRP 338, and LEU 356, as shown in Figure 11G.

## 4. Discussion

The broad use of essential volatile oils from Curcumae Rhizoma in the treatment of many diseases, including tumors, has a long and storied history. While HCC is a fatal major malignance among liver cancers, previous studies have not yet explored the potential of curcumol against this form of the disease. Therefore, the present study attempted to make the first step to address this issue. As blindly conducting experiments on a large scale is both time and resource consuming, in silico method was employed in the present study in order to improve the effectiveness and save labor.

In the present study, out of 371 HCC samples and 276 normal tissue samples, 44 common targets were mined from various authorized databases (e.g., TCGA, GTEx, GeneCards, OMIM, TCD, etc.). With these targets, functional analyses were carried out to gain a macroscopic understanding of their roles against HCC. It appears that curcumol could function by inducing apoptosis and inflammation, disturbing vessel generation of the tumor. Notably, VEGFA appears to be down-regulated so that vascularization of tumor tissue can be suppressed. These findings suggest that curcumol may function against HCC via inducing cell death and immunological reactions, as well as disturbing intra-tumor vascularization [52] and eliminating adverse effects of currently used drugs.

Moreover, a preliminary PPI network was also constructed, trying to gain in-depth insight into their functions. Via topological optimization, we conducted functional clustering for the preliminary PPI network. Each cluster was followed by GO terms and KEGG pathways enrichment analyses through which we found that proteins in cluster 1 were mainly associated with apoptosis, whereas proteins in cluster 2 were mainly associated with neurological signaling. Moreover, a core PPI subnetwork with nine key nodes was extracted, which was used to screen key targets in the following analyses.

In addition, we conducted a survival analysis for different types of survival (i.e., OS, PFS, DFS, DSS) for the 44 common targets where 17 genes were considered as significantly prognosis related.

Among them, 2 key targets, SQSTM1 and RELA, were sorted out by intersecting between the 9 key targets and these 17 significantly prognosis-related genes. From the aspects of cancer grading and Spearman correlation analysis, we provided a brief summary of RELA and SQSTM1, which emphasized their importance in HCC with ROC curve plotted to evaluate the reliability of our analyses. Then, we conducted in situ analysis by using the histological slides from HCC tissues and normal tissues. It was found that both RELA and SQSTM1 showed relatively low expression in normal tissues, while in the histological slides from HCC patients, they exhibited high expression levels, indicating that our computational prediction is in suitable agreement with the observation results. Consequently, it was found that their overexpression is statistically significant in tumor tissues, and they themselves are correlated with poor prognosis.

Via molecular docking, we found RELA and SQSTM1 showed high affinity to the curcumol molecule and are thus thought to be the key players in the potential anti-HCC mechanism of curcumol. Therefore, we further verified the stability of their binding by performing molecular dynamic simulation to ensure the binding was not affected by different docking protocols. As a result, we found that both the RELA-curcumol complex and SQSTM1-curcumol complex were stable during 100 ns-long molecular dynamic simulations. Combined with current literature, it is believed that curcumol decreases the expression level of RELA [53] and has certain indirect interactions with SQSTM1 expression, though whether these interactions may result in up-regulation or down-regulation remains unclear [52].

Although further experiments are needed, the present study elucidated the potential of curcumol against HCC, providing an important basis for in vivo studies. The approach described in the current work could also be used to screen for other biologically active compounds used in traditional Eastern medicine.

## 5. Conclusions

In summary, the present study demonstrated the potential underlying mechanism of curcumol in HCC treatment based on a network pharmacology method. We revealed that curcumol seemed to play a critical role in HCC by affecting multiple biotargets. Moreover, our molecular docking and molecular dynamic simulation results also suggested that the RELA and SQSTM1 proteins could bind with curcumol molecule stably, providing an important basis for further investigation, although this study also has certain limitations as pharmacological and clinical research still need to further validate our findings.

## Figures and Tables

**Table 1:** Pharmacological and molecular properties of curcumol. MW: molecular weight; Hdon: hydrogen donor; Hacc: hydrogen acceptor; AlogP: partition coefficient of octanol to water; RBN: number of bonds that can perform free rotation; OB: oral bioavailability; Caco2: Caco2 permeability; BBB: blood-brain barrier; DL: drug likeliness; TPSA: surface sum over all polar atoms, primarily oxygen and nitrogen, also including their attached hydrogens.

Name	MW	Hdon	Hacc	AlogP	RBN	OB	Caco2	BBB	DL	TPSA
Curcumol	236.39 Da	1	2	2.79	1	103.55%	1.12 nm/s	1.23	0.13	0.25 Å^2^

## Data Availability

The datasets presented in this study can be found in online repositories. The names of the repository/repositories and accession number(s) can be found in the article/Supplementary Material.

## Supplementary Materials

The following supporting information can be downloaded at: https://www.mdpi.com/article/10.3390/genes13040653/s1, File S1: Expression distribution of common targets, File S2: GSEA result of common targets, File S3: Rare data for top 10 most enriched GO terms in biological process, cellular component, and molecular function of common targets, File S4: Rare data for KEGG enrichment result of common targets, File S5: Rare data for cluster classification performed by MCODE, File S6: Rare data for GO terms and KEGG pathways enrichment analyses of cluster 1 and cluster 2, File S7: Prognosis-related genes, Table S1: Putative targets of curcumol.
