# Supplementary material for: Network Pharmacology-Based Approach Combined with Bioinformatic Analytics to Elucidate the Potential of Curcumol against Hepatocellular Carcinoma
Source: Genes (Basel). 2022 Apr 7;13(4):653. doi: 10.3390/genes13040653 (PMC9028201; doi:10.3390/genes13040653)
Supplement: Supplementary file 1 [file genes-13-00653-s001.zip › Table S1_Putative targets of curcumol .pdf]

Table S1 Putative targets of curcumol

Num.	Gene Symbol	Description
1	VEGFA	vascular endothelial growth factor A
2	KLF5	kruppel like factor 5
3	ATG5	autophagy related 5
4	KDR	kinase insert domain receptor
5	POSTN	periostin
6	ANGPT2	angiopoietin 2
7	GPT	glutamic--pyruvic transaminase
8	HIF1A	hypoxia inducible factor 1 subunit alpha
9	SQSTM1	sequestosome 1
10	CD34	CD34 molecule
11	PECAM1	platelet and endothelial cell adhesion molecule 1
12	VWF	Von Willebrand factor
13	ICAM1	intercellular adhesion molecule 1
14	TNF	tumor necrosis factor
15	VCAM1	vascular cell adhesion molecule 1
16	ATG12	autophagy related 12
17	ATG7	autophagy related 7
18	BECN1	beclin 1
19	IL18	interleukin 18
20	IL6	interleukin 6
21	MAP1LC3B	microtubule associated protein 1 light chain 3 beta
22	MTOR	mechanistic target of rapamycin kinase
23	NLRP3	NLR family pyrin domain containing 3
24	PRKAB1	protein kinase AMP-activated non-catalytic subunit beta 1
25	RELA	RELA proto-oncogene, NF-kB subunit
26	ULK1	unc-51 like autophagy activating kinase 1
27	SLC6A3	dopamine transporter (by homology)
28	SIGMAR1	sigma opioid receptor
29	SLC6A2	norepinephrine transporter
30	SLC6A4	serotonin transporter
31	PARP1	poly [ADP-ribose] polymerase-1
32	MDM2	p53-binding protein Mdm-2
33	TRPV3	transient receptor potential cation channel subfamily V member 3
34	CHRM4	muscarinic acetylcholine receptor M4
35	CHRM2	muscarinic acetylcholine receptor M2
36	OPRM1	mu opioid receptor
37	OPRD1	delta opioid receptor
38	OPRK1	kappa Opioid receptor
39	ADRA2C	adrenergic receptor alpha 2
40	HSD17B2	Estradiol 17-beta-dehydrogenase 2
41	NR3C1	glucocorticoid receptor

42	PTK2B	protein tyrosine kinase 2 beta
43	IDO1	indoleamine 2,3-dioxygenase
44	KCNA5	voltage-gated potassium channel subunit Kv1.5
45	P2RX7	P2X purinoceptor 7
46	NR3C2	mineralocorticoid receptor
47	PGR	progesterone receptor
48	HCRTR2	Orexin receptor 2
49	HCRTR1	Orexin receptor 1
50	HRH3	Histamine H3 receptor
51	HRH4	Histamine H4 receptor

T1_Putative targets of curcumol molecule. Those in red under the Gene Symbol column are the common targets.
