# Supplementary figures and images for: Network Pharmacology-Based Approach Combined with Bioinformatic Analytics to Elucidate the Potential of Curcumol against Hepatocellular Carcinoma
Source: Genes (Basel). 2022 Apr 7;13(4):653. doi: 10.3390/genes13040653 (PMC9028201; doi:10.3390/genes13040653)

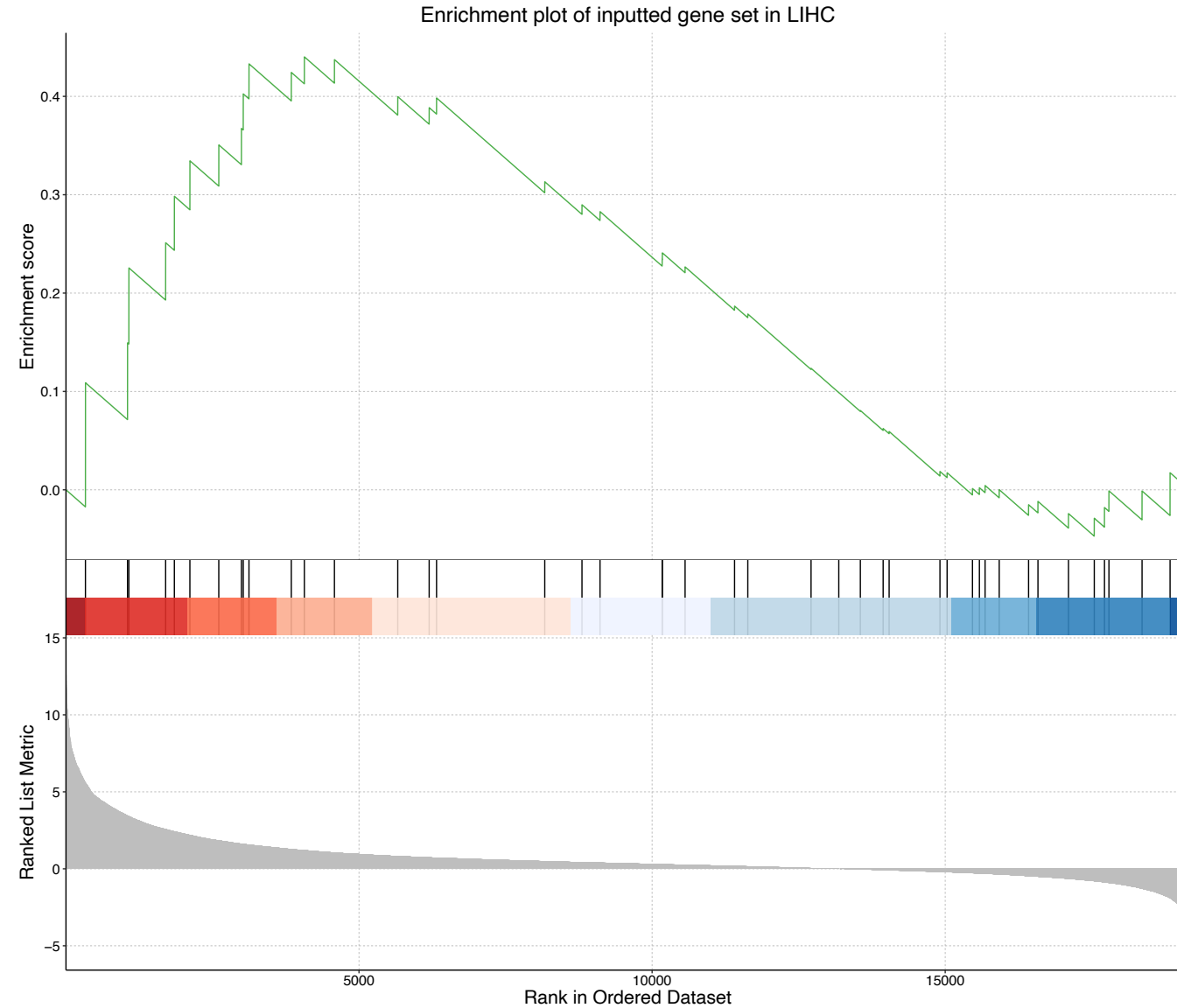

| Cancer type | ES   | NES  | P value | P adj. |
|-------------|------|------|---------|--------|
| LIHC        | 0.44 | 1.05 | 0.41    | 0.41   |

Supplement: Supplementary file 1 [file genes-13-00653-s001.zip › File S2_GSEA result of common targets .pdf]
