# Supplementary material for: Network Pharmacology-Based Approach Combined with Bioinformatic Analytics to Elucidate the Potential of Curcumol against Hepatocellular Carcinoma
Source: Genes (Basel). 2022 Apr 7;13(4):653. doi: 10.3390/genes13040653 (PMC9028201; doi:10.3390/genes13040653)
Supplement: Supplementary file 1 [file genes-13-00653-s001.zip › File S1_Expression distribution of common targets_.zip!/File S1_Expression distribution of common targets_/Expression distribution_1.pdf]

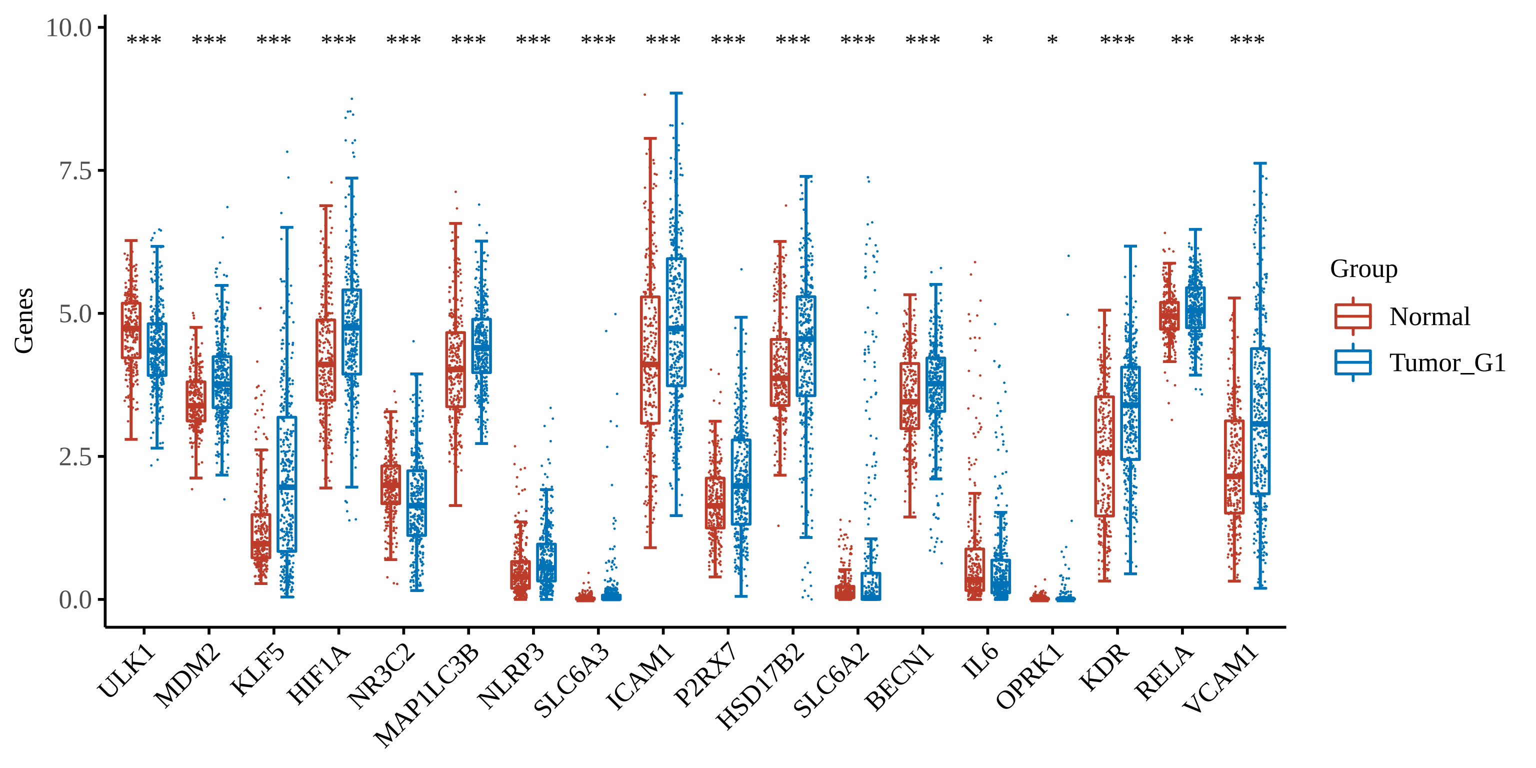

10.0
7.5
5.0
2.5
0.0
Genes
*** *** *** *** *** *** *** *** *** *** *** *** *** * * *** ** ***
ULK1
MDM2
KLF5
HIF1A
NR3C2
MAP1LC3B
NLRP3
SLC6A3
ICAM1
P2RX7
HSD17B2
SLC6A2
BECN1
IL6
OPRK1
KDR
RELA
VCAM1
Group
Normal
Tumor_G1
