# Supplementary material for: Network Pharmacology-Based Approach Combined with Bioinformatic Analytics to Elucidate the Potential of Curcumol against Hepatocellular Carcinoma
Source: Genes (Basel). 2022 Apr 7;13(4):653. doi: 10.3390/genes13040653 (PMC9028201; doi:10.3390/genes13040653)
Supplement: Supplementary file 1 [file genes-13-00653-s001.zip › File S1_Expression distribution of common targets_.zip!/File S1_Expression distribution of common targets_/Expression distribution_2.pdf]

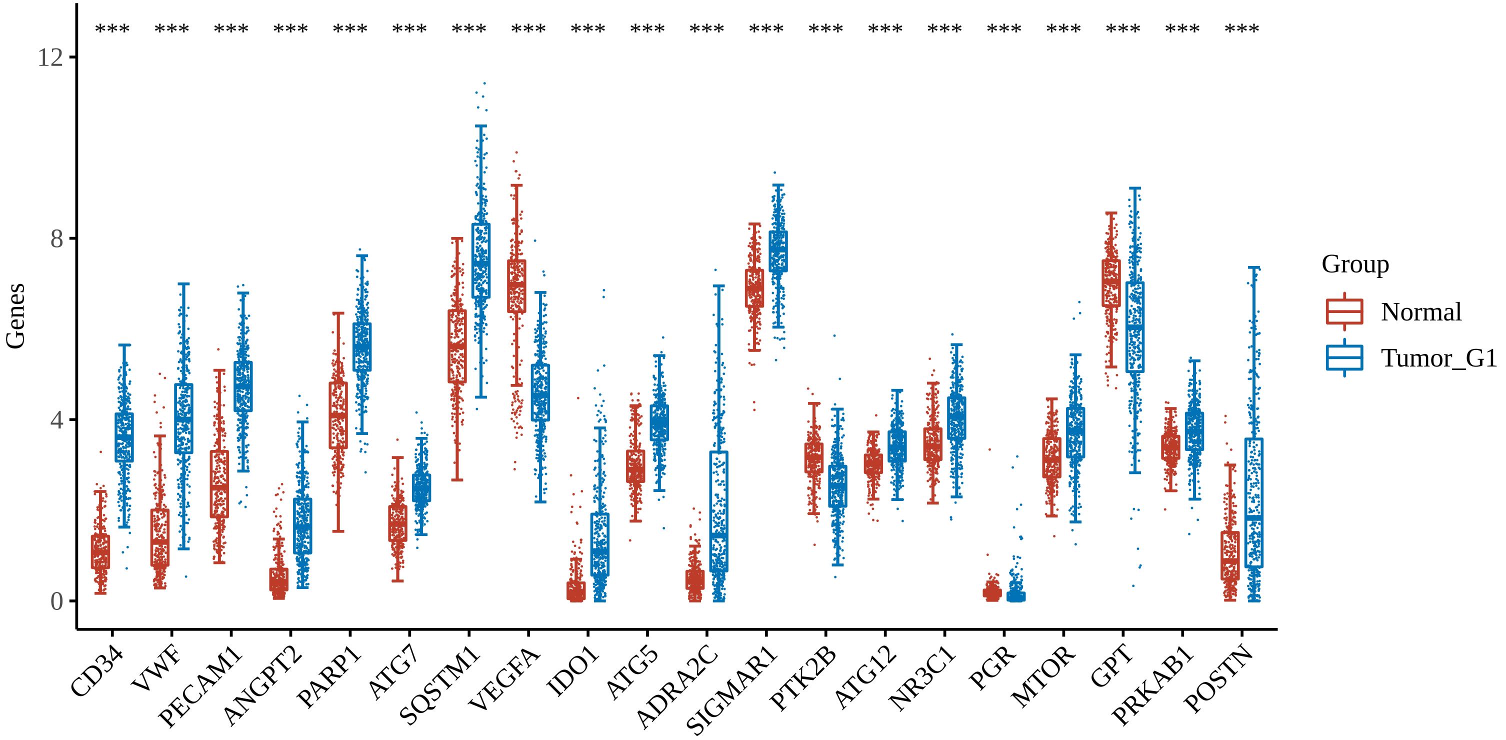

*** *** *** *** *** *** *** *** *** *** *** *** *** *** *** *** *** *** *** ***
Genes
0
4
8
12
CD34
VWF
PECAM1
ANGPT2
PARP1
ATG7
SQSTM1
VEGFA
IDO1
ATG5
ADRA2C
SIGMAR1
PTK2B
ATG12
NR3C1
PGR
MTOR
GPT
PRKAB1
POSTN
Group
Normal
Tumor_G1
