# Supplementary material for: Network Pharmacology-Based Approach Combined with Bioinformatic Analytics to Elucidate the Potential of Curcumol against Hepatocellular Carcinoma
Source: Genes (Basel). 2022 Apr 7;13(4):653. doi: 10.3390/genes13040653 (PMC9028201; doi:10.3390/genes13040653)
Supplement: Supplementary file 1 [file genes-13-00653-s001.zip › File S7_Prognosis related genes_.zip!/File S7_Prognosis related genes_/KM curves/KM curves_DSS.zip!/Θ1⁄2ÿμ╕àτƒóΘçÅσ¢╛/VEGFA_Fig.pdf]

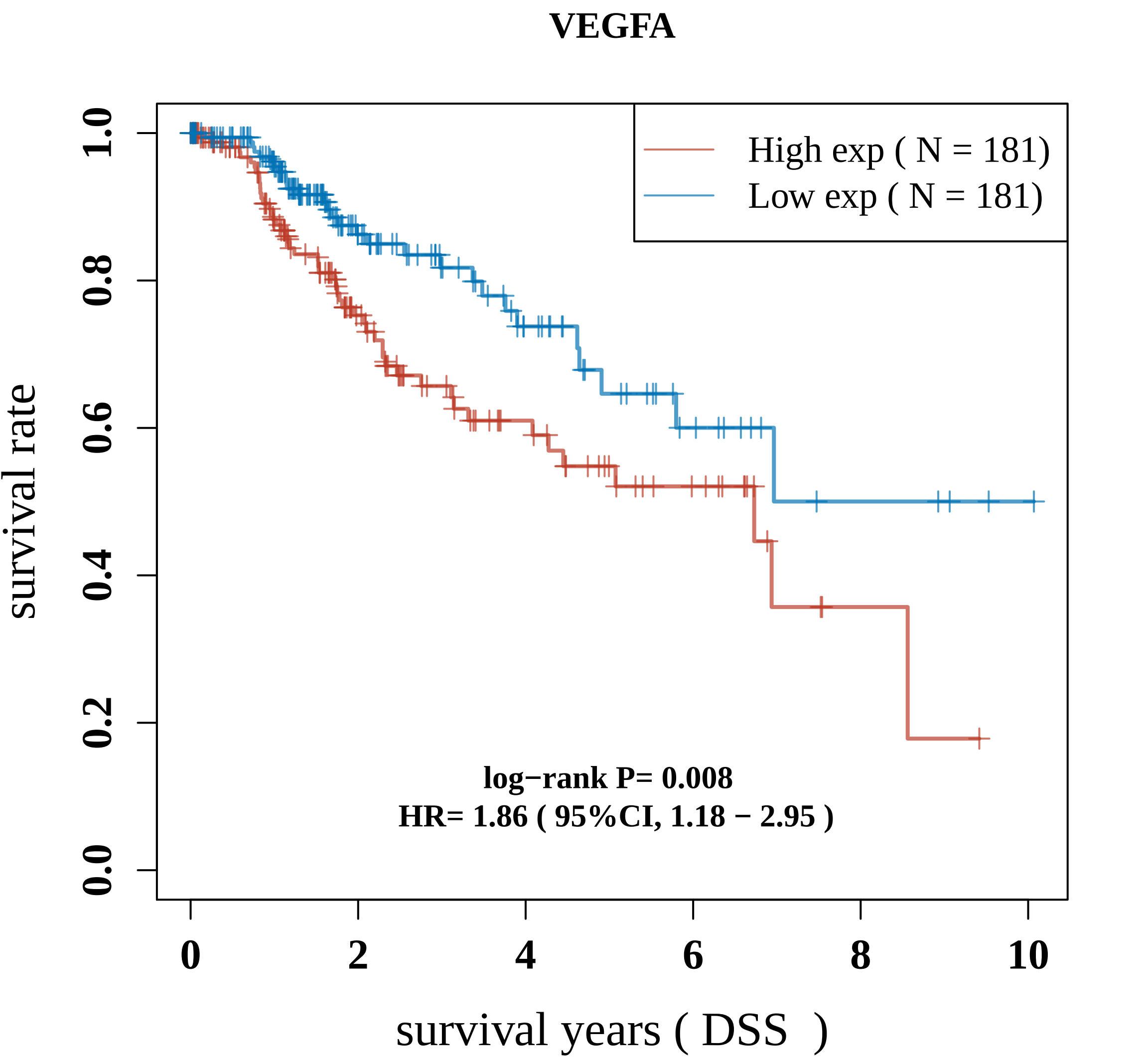
VEGFA
survival rate
1.0
0.8
0.6
0.4
0.2
0.0
High exp ( N = 181)
Low exp ( N = 181)
log−rank P= 0.008
HR= 1.86 ( 95%CI, 1.18 − 2.95 )
0
2
4
6
8
10
survival years ( DSS )
