# Supplementary material for: Network Pharmacology-Based Approach Combined with Bioinformatic Analytics to Elucidate the Potential of Curcumol against Hepatocellular Carcinoma
Source: Genes (Basel). 2022 Apr 7;13(4):653. doi: 10.3390/genes13040653 (PMC9028201; doi:10.3390/genes13040653)
Supplement: Supplementary file 1 [file genes-13-00653-s001.zip › File S7_Prognosis related genes_.zip!/File S7_Prognosis related genes_/KM curves/KM curves_OS.zip!/ANGPT2_Fig.pdf]

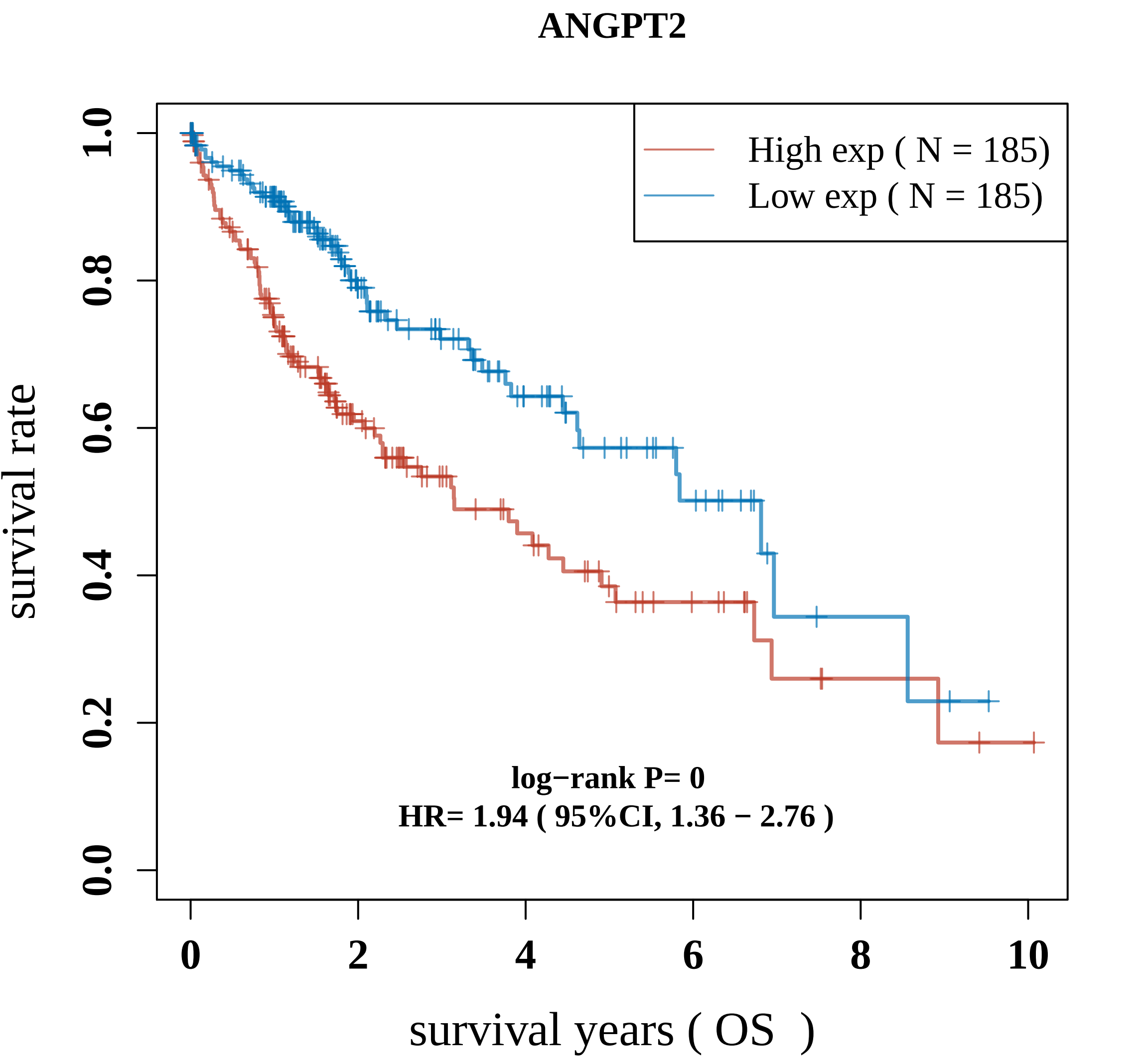
ANGPT2
High exp (N = 185)
Low exp (N = 185)
log−rank P= 0
HR= 1.94 (95%CI, 1.36 − 2.76)
survival rate
survival years (OS)
0.0
0.2
0.4
0.6
0.8
1.0
0
2
4
6
8
10
