# Supplementary material for: Network Pharmacology-Based Approach Combined with Bioinformatic Analytics to Elucidate the Potential of Curcumol against Hepatocellular Carcinoma
Source: Genes (Basel). 2022 Apr 7;13(4):653. doi: 10.3390/genes13040653 (PMC9028201; doi:10.3390/genes13040653)
Supplement: Supplementary file 1 [file genes-13-00653-s001.zip › File S7_Prognosis related genes_.zip!/File S7_Prognosis related genes_/KM curves/KM curves_OS.zip!/HIF1A_Fig.pdf]

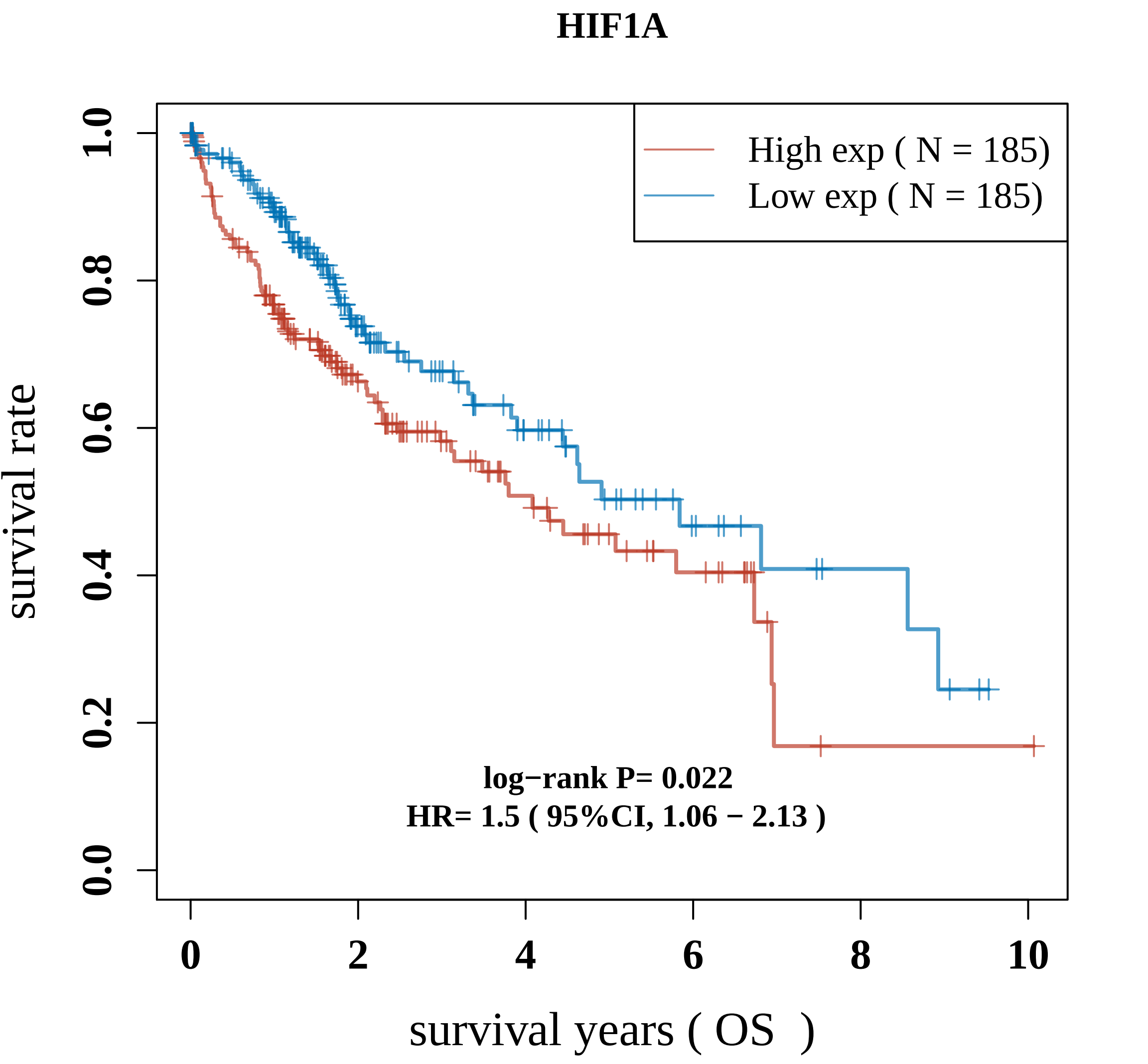

HIF1A
High exp (N = 185)
Low exp (N = 185)
log−rank P= 0.022
HR= 1.5 (95%CI, 1.06 − 2.13)
survival rate
1.0
0.8
0.6
0.4
0.2
0.0
0
2
4
6
8
10
survival years (OS)
